# Phosphatase ABI1 and okadaic acid-sensitive phosphoprotein phosphatases inhibit salt stress-activated SnRK2.4 kinase

**DOI:** 10.1186/s12870-016-0817-1

**Published:** 2016-06-13

**Authors:** Ewa Krzywińska, Maria Bucholc, Anna Kulik, Arkadiusz Ciesielski, Małgorzata Lichocka, Janusz Dębski, Agnieszka Ludwików, Michał Dadlez, Pedro L. Rodriguez, Grażyna Dobrowolska

**Affiliations:** Institute of Biochemistry and Biophysics, Polish Academy of Sciences, Pawińskiego 5a, 02-106 Warsaw, Poland; Present address: Nencki Institute of Experimental Biology, Polish Academy of Sciences, Pasteur 3, 02-093 Warsaw, Poland; Present address: Department of Chemistry, Warsaw University, Pasteur 1, 02-093 Warsaw, Poland; Department of Biotechnology, Institute of Molecular Biology and Biotechnology, Faculty of Biology, Adam Mickiewicz University in Poznań, Umultowska 89, 61-614 Poznań, Poland; Institute of Genetics and Biotechnology, University of Warsaw, Pawińskiego 5a, 02-106 Warsaw, Poland; Instituto de Biología Molecular y Celular de Plantas, Consejo Superior de Investigaciones Científicas-Universidad Politécnica de Valencia, ES-46022 Valencia, Spain

**Keywords:** Salinity, Osmotic stress signaling, SNF1-related protein kinases 2, SnRK2, Phosphoprotein phosphatases, *Arabidopsis thaliana*, ABI1, PPP, PP2C

## Abstract

**Background:**

SNF1-related protein kinases 2 (SnRK2s) are key regulators of the plant response to osmotic stress. They are transiently activated in response to drought and salinity. Based on a phylogenetic analysis SnRK2s are divided into three groups. The classification correlates with their response to abscisic acid (ABA); group 1 consists SnRK2s non-activated in response to ABA, group 2, kinases non-activated or weakly activated (depending on the plant species) by ABA treatment, and group 3, ABA-activated kinases. The activity of all SnRK2s is regulated by phosphorylation. It is well established that clade A phosphoprotein phosphatases 2C (PP2Cs) are negative regulators of ABA-activated SnRK2s, whereas regulators of SnRK2s from group 1 remain unidentified.

**Results:**

Here, we show that ABI1, a PP2C clade A phosphatase, interacts with SnRK2.4, member of group 1 of the SnRK2 family, dephosphorylates Ser158, whose phosphorylation is needed for the kinase activity, and inhibits the kinase, both in vitro and in vivo. Our data indicate that ABI1 and the kinase regulate primary root growth in response to salinity; the phenotype of *ABI1* knockout mutant (*abi1td*) exposed to salt stress is opposite to that of the *snrk2.4* mutant. Moreover, we show that the activity of SnRK2s from group 1 is additionally regulated by okadaic acid-sensitive phosphatase(s) from the phosphoprotein phosphatase (PPP) family.

**Conclusions:**

Phosphatase ABI1 and okadaic acid-sensitive phosphatases of the PPP family are negative regulators of salt stress-activated SnRK2.4. The results show that ABI1 inhibits not only the ABA-activated SnRK2s but also at least one ABA-non-activated SnRK2, suggesting that the phosphatase is involved in the cross talk between ABA-dependent and ABA-independent stress signaling pathways in plants.

**Electronic supplementary material:**

The online version of this article (doi:10.1186/s12870-016-0817-1) contains supplementary material, which is available to authorized users.

## Background

Osmotic stress, caused by salinity and drought, is one of the major factors limiting plant growth. In order to survive plants have developed numerous defense mechanisms which require diverse signaling pathways for their initiation and regulation, both abscisic acid (ABA)-dependent as well as ABA-independent [1–3].

SnRK2 kinases are key elements in the plant response to osmotic stress and ABA signaling [4–8]. The *Arabidopsis thaliana* and *Oryza sativa* genomes each encode ten members of the SnRK2 family. The kinases (both from Arabidopsis and rice) were expressed in plant protoplasts and their activity was analyzed in response to different treatments. The results revealed that all SnRK2s, except Arabidopsis SnRK2.9, are activated in response to treatment with different osmolytes and some of them additionally in response to ABA [9, 10]. Based on a phylogenetic analysis SnRK2s have been divided into three groups. This classification overlaps with the discrimination based on their activation by ABA and their role in ABA-dependent and ABA-independent signaling processes. Group 1 consists of kinases which are not activated by exogenous ABA in the absence of osmotic stress (further referred to as ABA-non-activated), group 2—those, which are not activated by ABA or activated very weakly, and group 3—strongly activated by ABA [9, 10] (Additional file 1: Figure S1). Among the SnRK2 family, the role of kinases from group 3 (Arabidopsis SnRK2.2, SnRK2.3, and SnRK2.6) in the ABA-dependent osmotic stress transduction pathway is best characterized. Together with RCAR/PYR/PYL (RCAR, regulatory component of ABA receptor/PYR1, pyrabactin resistance 1/PYL, PYR1-like) ABA receptors and clade A PP2C phosphatases, they form the core of the ABA signaling network [11–16]. The kinases are involved in plant defense against water deficit stress and in ABA-dependent plant development. They regulate stress-responsive gene expression and stomatal closure by phosphorylation of various cellular substrates e.g., AREB/ABF transcription factors, guard cell ion channels and several others [17, 18]. Much less is known concerning the role of kinases from the two other groups of SnRK2. Group 2 SnRK2s are involved in drought stress responses [19, 20]. Although Arabidopsis SnRK2.7 and SnRK2.8 from group 2 were shown to be weakly activated by exogenous ABA, they are considered not to play a physiological role in ABA signaling, or that it is marginal [4, 20]. Moreover, rice SnRK2s from this group are not activated by ABA [10]. The kinases from group 1 are activated extremely rapidly by high osmoticum—*Nicotiana tabacum* osmotic stress-activated kinase (NtOSAK, in tobacco) and SnRK2.4 and SnRK2.10 (in Arabidopsis) are fully active as soon as after 1 min of plant or cell exposure to salt [21, 22]. SnRK2.4 and SnRK2.10 regulate root growth and its architecture under salinity [22]. The importance of the ABA-non-activated SnRK2s in plant tolerance to water deficit stress was unraveled by a study performed by Fujii et al. [7]. They showed that plants lacking functional kinases from both group 1 and 2 are more affected by osmotic stress than the *snrk2.2/2.3/2.6* triple mutant (impaired in ABA-activated SnRK2s), as judged by root growth and fresh weight assessment.

It is well established that reversible phosphorylation of specific Ser/Thr residues in the SnRK2 activation loop is responsible for regulation of SnRK2s’ activity [10, 21, 23–25]. Recently Saruhashi et al. [26], showed that a kinase named ARK (for ABA and abiotic stress-responsive Raf-like kinase) acts upstream of SnRK2 in the moss *Physcomitrella patens*. Additionally, Arabidopsis glycogen synthase kinase 3 (GSK3)-like kinases phosphorylate and enhance the activity of SnRK2.2 and SnRK2.3 in response to ABA [27]. Beside upstream kinases, negative regulators (mainly phosphatases) control SnRK2 activity inside the cell. Some members of clade A PP2C protein phosphatases have been shown to dephosphorylate and in this way inactivate ABA-responsive SnRK2s (from group 3) [28, 29]. The discovery of soluble ABA receptors—RCAR/PYR/PYL—as cellular partners of the clade A PP2Cs [30–35] allowed building of a model of ABA signal transduction with RCAR/PYR/PYL, PP2Cs and SnRK2s forming the core of the signaling network [11–16]. In this model a PP2C phosphatase from clade A [e.g., ABA-insensitive 1 (ABI1), ABI2, hypersensitive to ABA 1 (HAB1) or PP2CA/ABA-hypersensitive germination 3 (PP2CA/AHG3)] interacts with an ABA-responsive SnRK2 (from group 3; Arabidopsis SnRK2.2, SnK2.3 and SnRK2.6) independently of ABA presence. In the absence of ABA, the phosphatase dephosphorylates the SnRK2, keeping it in an inactive form. The situation changes dramatically in stress conditions causing a rise in ABA level. Upon ABA binding RCAR/PYR/PYL undergoes major conformational changes and forms a complex with PP2C, thereby blocking its substrate-binding site. In this way SnRK2 is released from inhibition by PP2C and can be activated [36, 37]. The mechanism of the SnRK2 activation has been established only for the response to ABA. Some data indicate that also group 2 kinases (Arabidopsis SnRK2.7 and SnRK2.8) can be regulated by group A PP2C phosphatases [28], although their inactivation by those phosphatases has not been proven so far. In the case of the SnRK2s non-activated in response to ABA (group 1), the phosphatases involved in their regulation remain unknown. Clade A PP2Cs have not been considered to be such regulators since no interaction between SnRK2.10 (SnRK2 from group 1) and the phosphatases could be detected in yeast two-hybrid assays [25, 28], and the kinase activation in response to osmotic stress was the same in Arabidopsis PP2C mutants and wild type plants [25]. However, structural and some in vitro studies have not excluded the possibility that clade A PP2Cs could in principle dephosphorylate ABA-unresponsive SnRK2s [38].

Here, we describe studies undertaken to identify the phosphatases(s) regulating the activity of ABA-non-activated SnRK2s represented by Arabidopsis SnRK2.4 kinase. Our results show that ABI1, a member of the PP2C family, interacts with and inhibits SnRK2.4 in plant cells. Additionally, we show that SnRK2s, at least those that are not activated in response to ABA (from group 1), are inhibited in plant cells by okadaic acid-sensitive phosphatases from the PPP family.

## Results

### Analysis of interactions between Arabidopsis clade A PP2C phosphatases and SnRK2 kinases

*Nicotiana tabacum* osmotic stress-activated kinase (NtOSAK, GenBank: AAL89456) is a member of group 1 of the SnRK2 family, exhibiting highest sequence similarity to SnRK2.4 [TAIR: At1g10940] and SnRK2.10 [TAIR: At1g60940] in Arabidopsis ([39] and Additional file 1). Our studies indicated that NtOSAK activity is regulated by phosphorylation [21, 40], and the kinase is inactivated in vitro by dephosphorylation catalyzed by NtPP2C2 [GenBank: AB110956] phosphatase, member of the clade A of the PP2C family (Additional file 2). NtPP2C2 dephosphorylates specifically Ser154 and S158, whose phosphorylation is crucial for the kinase activity (Additional files 2 and 7 and [21]). These results suggested that clade A PP2Cs could regulate not only the ABA-activated but also other types of SnRK2s. Moreover, supplemental materials presented by Nishimura et al. [41] showed several peptides of SnRK2.4 and also of other SnRK2s from group 1 among peptides of proteins co-immunoprecipitating with ABI1 [TAIR: At4g26080], a PP2C clade A phosphatase.

**Fig. 1:**
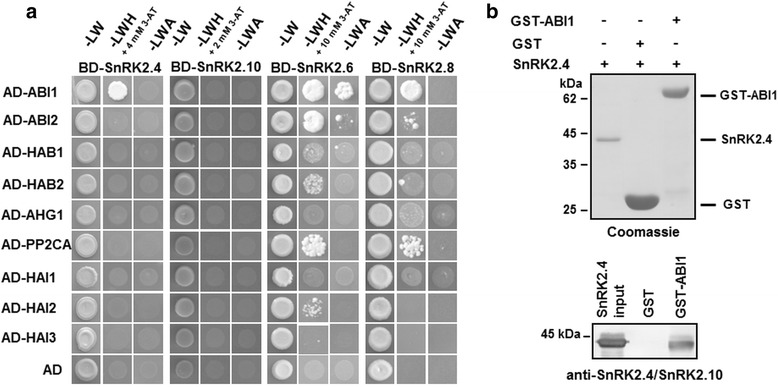
Analysis of interactions of selected members of the SnRK2 family with clade A PP2C phosphatases. **a** Yeast two-hybrid analysis; growth of yeast expressing the indicated constructs was monitored on selective media: without Leu and Trp (−LW); without Leu, Trp and His (−LWH) supplemented with different concentrations of aminotriazole (3-AT); without Leu, Trp and Ade (−LWA). AD, Gal4 activation domain; BD, Gal4 binding domain. Data represent one of three independent experiments showing similar results. **b** Pull down assays; GST-fused ABI1 or GST alone, bound to glutathione-sepharose beads, were incubated with recombinant SnRK2.4. Proteins bound to the resin were separated by SDS-PAGE, electroblotted to a membrane and visualized by immunoblotting using anti-SnRK2.4/SnRK2.10 antibodies. In parallel, visualization of proteins in samples used for the Western blotting was performed by staining with Coomassie Brilliant Blue, CBB. Data represent one of two independent experiments showing similar results

Therefore, using the yeast two-hybrid (YTH) system, we looked for interactions between ABA-non-activated SnRK2s, SnRK2.4 and SnRK2.10, which exhibit highest sequence similarity to NtOSAK (Additional file 1), and Arabidopsis PP2C clade A phosphatases. Additionally, to compare our results with published data, we assayed SnRK2s whose binding with PP2Cs had already been established—SnRK2.8 [TAIR: At1g78290] (from group 2) and SnRK2.6 [TAIR: At4g33950] (from group 3). Our results largely confirmed the previously published data [25, 28]. In our hands SnRK2.8 interacted in a clear-cut manner with two phosphatases, ABI1 and PP2CA [TAIR: At3g11410], and weakly with ABI2 [TAIR: At5g57050] (Fig. 1a). Similarly to data presented by Umezawa et al. [28] and Vlad et al. [25] we did not see an interaction of SnRK2.10 (from group 1) with any of the phosphatases studied. However, for SnRK2.4, we did observe reproducible interaction with one of the phosphatases, ABI1 (Fig. 1a). The interaction was weaker than that observed for SnRK2.6 or SnRK2.8; the growth of yeast expressing SnRK2.4 in fusion with the Gal4 DNA-binding domain and ABI1 in fusion with the activation domain was abolished on medium supplemented with aminotriazole at concentration higher than 4 mM.

To verify the observed interaction between ABI1 and SnRK2.4 in vitro pull-down assays were performed. Recombinant glutathione-sepharose-bound ABI1 in fusion with GST, or GST alone (as a negative control) were used as bait. The presence of bound SnRK2.4 was analyzed by immunoblotting using antibodies against the N-terminal peptide specific for SnRK2.4 and SnRK2.10 (anti-SnRK2.4/SnRK2.10). As shown in Fig. 1b SnRK2.4 interacted with ABI1 in the pull-down assay.

### ABI1 interacts with SnRK2.4 *in planta*

**Fig. 2:**
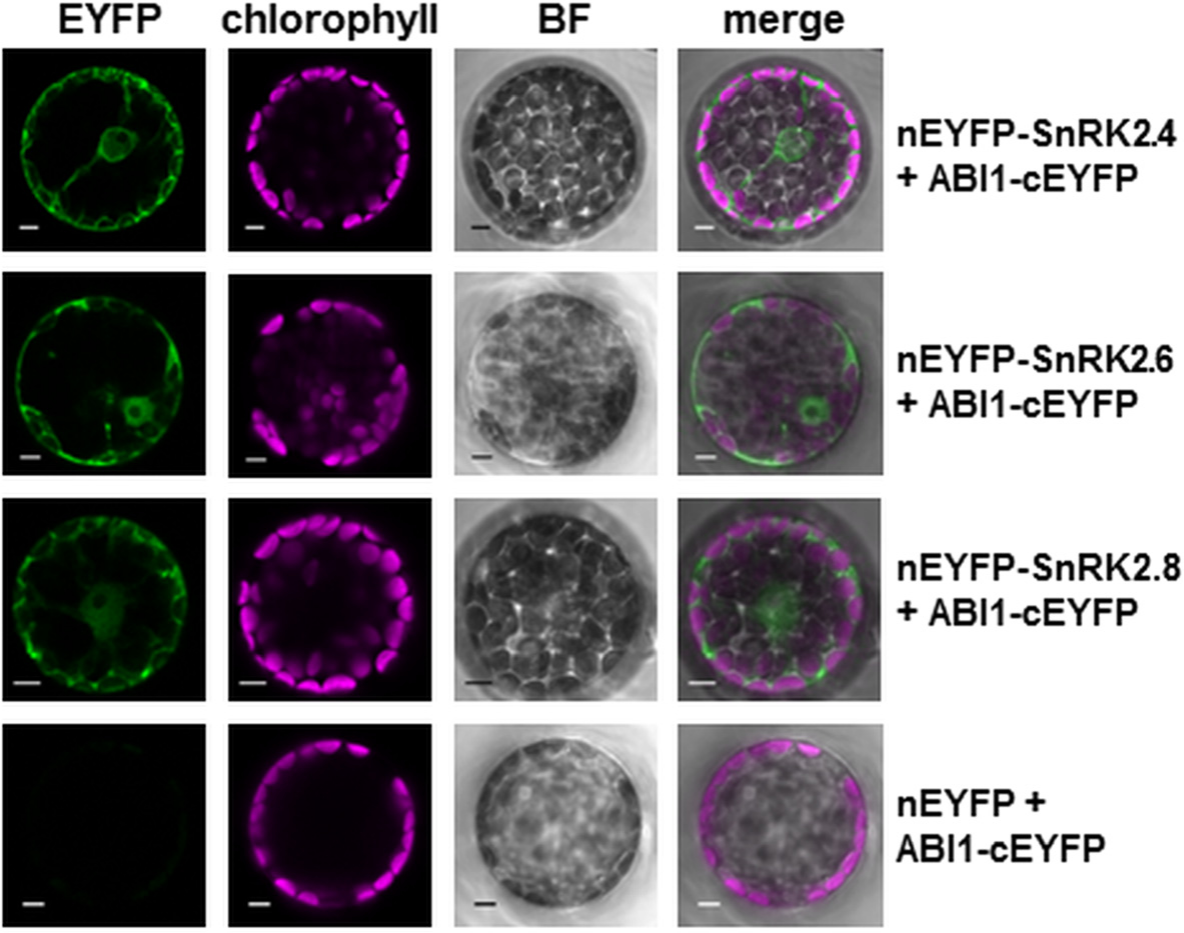
ABI1 interacts with SnRK2.4 *in planta*. Interaction of the proteins was analyzed by BiFC assay. Protoplasts isolated from Arabidopsis leaves were transiently co-transformed with pairs of plasmids encoding: ABI1-cEYFP and nEYFP-SnRK2.4 or ABI1-cEYFP and nEYFP-SnRK2.8 Positive control of the BiFC assay is provided by the ABI1-cEYFP/nEYFP-SnRK2.6 interaction. For negative control, cEYFP-ABI1 was co-expressed with nEYFP. Scale bar = 10 μm; BF, bright field. Data represent one of three independent experiments showing similar results

To investigate whether the interaction between SnRK2.4 and ABI1 takes place *in planta* we resorted to the bimolecular fluorescence complementation (BiFC) approach. Additionally, we tested the interaction between ABI1 and SnRK2.8, which had never been studied for SnRK2 kinases from group 2 in plant cells. SnRK2.4 or SnRK2.8 or SnRK2.6 (as a positive control) together with ABI1, each fused to complementary non-fluorescent fragments of YFP, were transiently produced in Arabidopsis protoplasts. We observed interactions between ABI1 and all the kinases studied both in the nucleus and in the cytoplasm (Fig. 2).

### ABI1 dephosphorylates and inhibits SnRK2.4 activity both in vitro and in plant cells

**Fig. 3:**
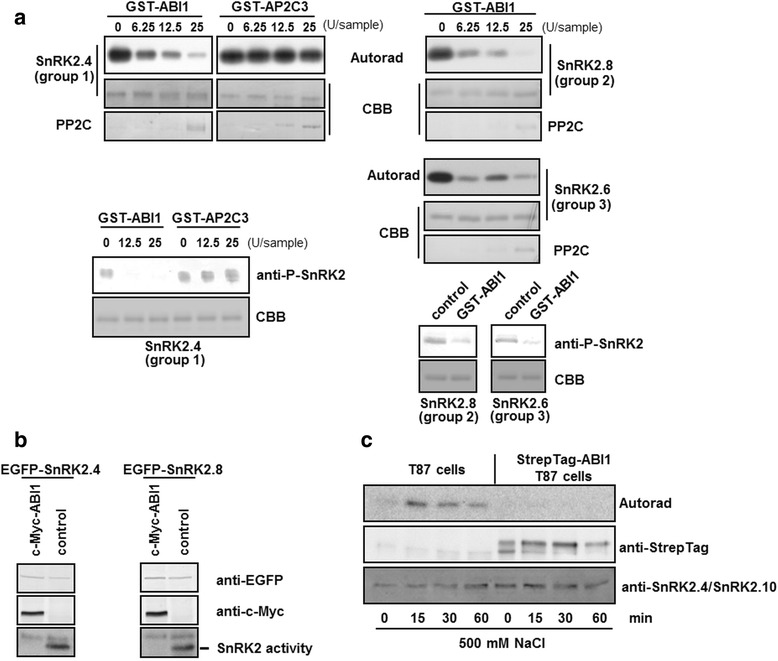
ABI1 inhibits kinases from all SnRK2 groups in vitro and *in planta*. **a** Recombinant SnRK2.4 (1 μg) was incubated with increasing amounts of PP2Cs for 30 min at 30 °C and kinase activity was analyzed by in-gel kinase assay using MBP as substrate. Dephosphorylation of SnRK2.4 was monitored by immunoblotting using specific anti-P-SnRK2 antibodies. Recombinant kinases SnRK2.8 and SnRK2.6 (as positive control) were incubated with increasing amounts of phosphatases for 30 min at 30 °C and the kinase activity was monitored by in–gel kinase assay with MBP as substrate. SnRK2 phosphorylation status was visualized by immunoblotting with specific anti-P-SnRK2 antibodies recognizing a specific phosphorylated residue in the kinase activation loop (Ser-158 in SnRK2.4, Ser-175 in SnRK2.6, and Thr-158 in SnRK2.8). In parallel, visualization of proteins in samples used for the Western blotting was performed by staining with Coomassie Brilliant Blue, CBB. Data represent one of two independent experiments showing similar results. **b** EGFP-SnRK2s were co-expressed with c-Myc-ABI1 in Arabidopsis protoplasts isolated from the T87 cell line. The protein level of kinases studied and ABI1 in extracts from protoplasts treated with 300 mM NaCl was monitored by immunoblotting; the activity of the kinases was analyzed by in-gel kinase activity assay using MBP as substrate. **c** Arabidopsis T87 cells and T87 cells expressing StrepTag-ABI1 were exposed to 500 mM NaCl. Activity of SnRK2.4/SnRK2.10 in the cell extracts was monitored by immunocomplex kinase activity assay, using MBP as substrate. The level of StrepTag-ABI1 and SnRK2.4/SnRK2.10 proteins was monitored by immunoblotting. Autorad, autoradiograph; CBB, Coomassie Brilliant Blue

In order to check whether ABI1 is able to regulate SnRK2.4 we analyzed the effect of the recombinant phosphatase on the kinase activity. In parallel, to evaluate the impact of a phosphatase from a group other than clade A PP2C on SnRK2.4 in these conditions, AP2C3 [TAIR: At2g40180], a clade B PP2C, was also assayed. The specific activity of the recombinant phosphatases was estimated using a commercial phosphatase activity assay (Additional file 3). Equal amounts of activity of each of the phosphatases were then used in in vitro assays against SnRK2.4 as substrate. ABI1 inhibited the kinase (Fig. 3a) and significantly reduced phosphorylation of Ser-158, a conserved residue in the activation loop of SnRK2s, phosphorylation of which is crucial for their activity [21, 23, 24]. In contrast, AP2C3 did not affect the phosphorylation status of Ser-158 or the kinase activity. Additionally, the effect of ABI1 on SnRK2.8 (from group 2) and SnRK2.6 (from group 3, as a positive control) was studied. The phosphatase dephosphorylated and inhibited the kinases (Fig. 3a). GST alone had no influence on the activity of the SnRK2s studied (Additional file 4). The results suggested that ABI1 is the regulator of various SnRK2 kinases, including the ABA-non-activated ones.

In order to investigate whether ABI1 dephosphorylates and inactivates SnRK2.4 *in planta* the kinase, in fusion with EGFP, was transiently expressed alone or co-expressed with c-Myc-ABI1 in Arabidopsis protoplasts. Transformed protoplasts were subjected to salinity stress (300 mM NaCl) for 20 min and the activity of the kinase was monitored by in-gel kinase assay. This experiment revealed that ABI1 inactivates SnRK2.4 *in planta* (Fig. 3b). Similarly, co-expression of ABI1 with SnRK2.8 caused inhibition of the kinase activity under salt stress (Fig. 3b).

We also monitored the activity of ABA-non-activated SnRK2 kinases in Arabidopsis T87 cells expressing StrepTag-ABI1 [42]. The cells were treated with 500 mM NaCl for various times and the activity of SnRK2s immunoprecipitated using anti-SnRK2.4/SnRK2.10 antibodies was monitored. Due to the specificity of the antibodies used, both SnRK2.4 and SnRK2.10 were immunoprecipitated from the cell extracts. As a consequence, their combined activity was analyzed, since they cannot be distinguished after SDS-PAGE separation. The salt-induced SnRK2.4/SnRK2.10 activity was significantly lowered (nearly undetectable) in cells expressing StrepTag-ABI1 in comparison to control T87 cells (Fig. 3c).

### Knockout insertion mutant *abi1td* exhibits opposite phenotype to *snrk2.4* in primary root growth of seedlings exposed to salinity stress

**Fig. 4:**
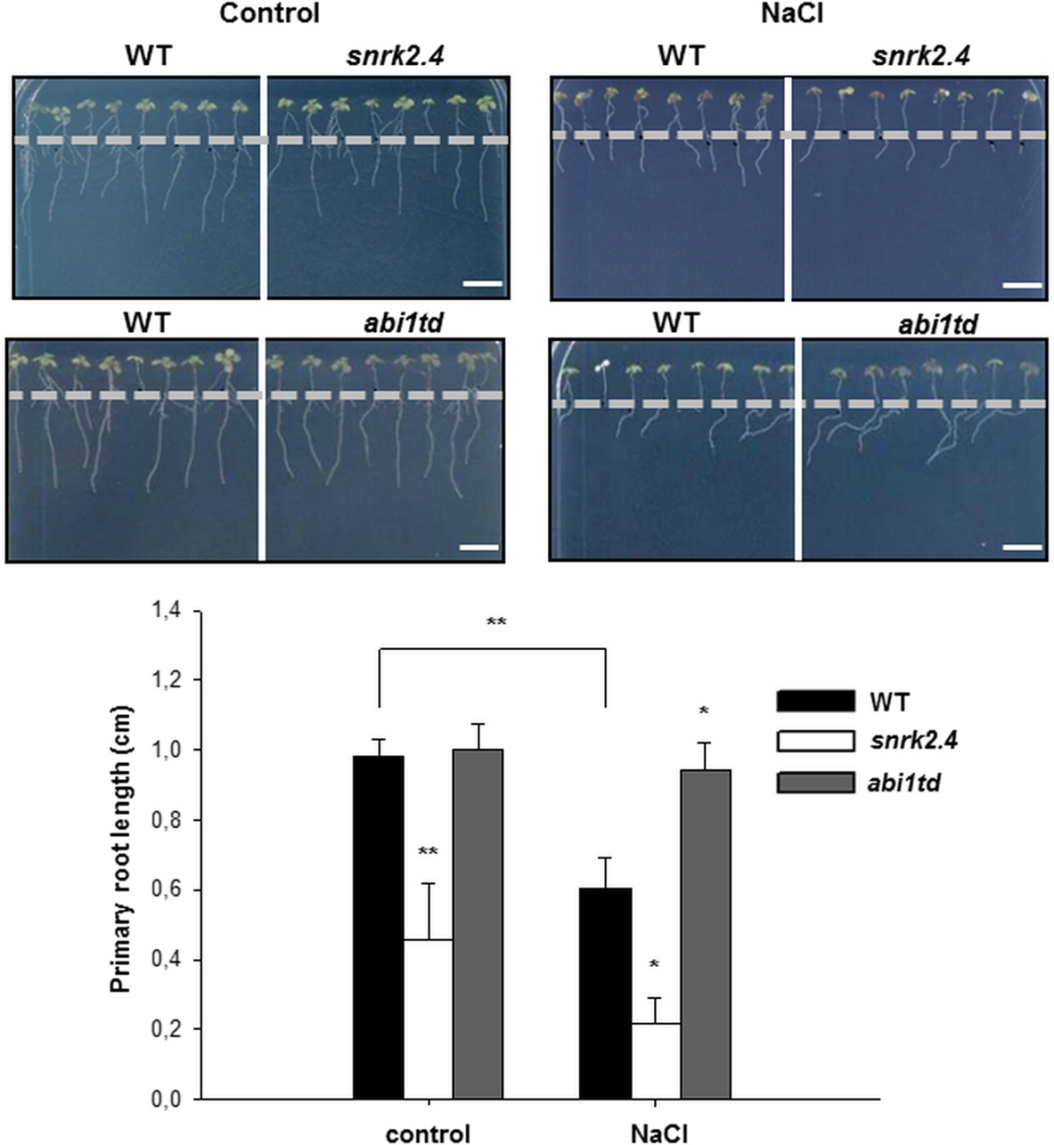
ABI1 negatively regulates root elongation under salt stress. Seven-day-old seedlings grown vertically on ½ MS media were transferred into square Petri plates with control media or 115 mM NaCl and the increase in primary root length was measured. Dotted line shows the approximate length of roots just after transfer. The graphs present mean values (± SE), n = 7. Statistical analysis was done by *t*-test; bar on photographs = 0.5 cm

In order to analyze physiological consequences of the regulation of SnRK2.4 activity by ABI1 phosphatase we compared phenotypes of plants with altered SnRK2.4 or ABI1 levels. As it was mentioned before SnRK2.4 and SnRK2.10 are involved in the regulation of root architecture in plants exposed to salinity stress; the primary root (PR) length of the *snrk2.4* mutant exposed to 115 mM NaCl is significantly shorter in comparison to wild type seedlings [22]. To check whether ABI1 is involved in the plant response to salinity we analyzed the phenotypes of *abi1td*, a mutant with T-DNA insertion in *ABI1*, and in parallel the *snrk2.4* mutant and wild type Col-0 (WT) plants. We compared the PR length of seedlings exposed to 115 mM NaCl (and also in control conditions). In our hands, in control conditions the roots of *snrk2.4* were shorter than those of the WT plants or the *abi1td* mutant (Fig. 4a and b). After stress application, the PRs of the *snrk2.4* mutant and WT seedlings grown on medium with salt were significantly shorter than the controls. However, the roots of *snrk2.4* were the most affected by salt, exhibiting the most severe root growth inhibition (Additional file 5) and being approximately 65 % shorter than the roots of WT seedlings. In contrast, the roots of the *abi1td* mutant were significantly longer in comparison to WT, by 40 % (Fig. 4a and b), indicating that ABI1 is involved in the regulation of root growth under salinity stress.

### Group 1 SnRK2s are regulated by okadaic acid-sensitive phosphatase(s)

**Fig. 5:**
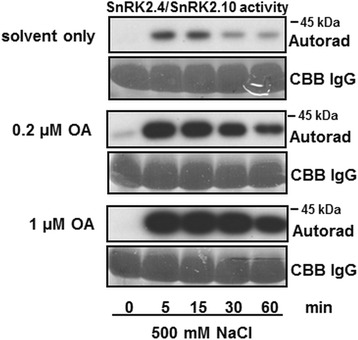
Pretreatment of T87 cells with OA enhances the salinity-induced activity of ABA-non-activated SnRK2s. Arabidopsis T87 cells were preincubated with indicated concentrations of OA for 2 h, followed by exposure to salinity stress. SnRK2.4/SnRK2.10 activity in cell extracts was monitored by immuno-in-gel kinase activity assay, using MBP as substrate and anti-SnRK2.4/SnRK2.10 antibodies. The level of IgG is presented as loading control. CBB, Coomassie Brilliant Blue. Data represent one of two independent experiments showing similar results

Our previous results showed that the catalytic subunit of maize phosphatase PP2A can dephosphorylate and inactivate NtOSAK [40]. Therefore, in order to test if SnRK2.4 could be regulated by phosphatases other than PP2Cs in plant cells, we monitored SnRK2.4/SnRK2.10 activity in Arabidopsis T87 cells exposed to okadaic acid (OA), an inhibitor of the PPP family of serine/threonine-specific phosphoprotein phosphatases (e.g., PP1 and PP2A) [43]. T87 cells were pretreated with 0.2 μM or 1 μM OA (or with the OA solvent only as a control) for 2 h and then exposed to 500 mM NaCl. SnRK2.4/SnRK2.10 activity in cells exposed to such treatment was measured by immunocomplex kinase activity assay. The results showed significantly higher SnRK2.4/SnRK2.10 activity in cells pretreated with OA in comparison to control, indicating that also OA-sensitive phosphatases limit the salinity-induced SnRK2 activity (Fig. 5).

## Discussion

SnRK2 kinases, especially those from group 1, are rapidly and transiently activated during the plant response to salinity stress or water deficit [19, 21, 22, 40, 44]. In contrast to the well-studied ABA-activated SnRK2s (group 3), the mode of regulation of SnRK2s from group 1 is unknown. We have shown that some members of group 1 are inhibited by clade A PP2C phosphatases. This result was intriguing for several reasons. First, no interaction between SnRK2.10 (SnRK2 from group 1, with high sequence similarity to SnRK2.4) and these phosphatases could be detected previously [25, 28]. Second, SnRK2.10 activation in response to osmotic stress was similar in Arabidopsis *pp2c* mutants and wild type plants [25]. Finally, group 1 SnRK2s are not involved in a direct ABA-dependent pathway, induced by ABA application [9, 24], in contrast to the current paradigm for clade A PP2Cs. To elucidate the regulation of group 1 SnRK2s, we took advantage of our previous unpublished data indicating that NtOSAK, the tobacco kinase, member of group 1 of the SnRK2 family, can be dephosphorylated and inactivated by NtPP2C2 (clade A PP2C from tobacco) and data mining in Nishimura et al. [41] showing co-immunoprecipitation of group 1 SnRK2s and ABI1. Next, we showed that SnRK2.4 interacts with ABI1 and is dephosphorylated and inhibited in vitro by the phosphatase, similarly to SnRK2.8 (group 2) and the ABA-activated SnRK2.6 (group 3). This probably reflects the fact that the catalytic domains (including activation loops) of all SnRK2s are nearly identical. Analysis of structures of the SnRK2.6-HAB1 [37] and SnRK2.6-ABI1 [45] complexes has revealed that the phosphatase and kinase active sites form the major binding interface, with three distinct regions in the kinase domain contributing to the kinase - phosphatase interaction. All the residues crucial for the complex formation with the phosphatases are also conserved in the SnRK2s from group 1. The C-terminal part (“Domain II”, specific for ABA-activated SnRK2s) seems to be involved mainly in complex stabilization.

Our results showed that inhibition of SnRK2.4 by ABI1 occurs both in vitro and *in planta*. In plant protoplast and in Arabidopsis T87 cells overexpressing ABI1 the activity of SnRK2.4 (or SnRK2.4/SnRK2.10 in plant cells) is significantly lower than in cells/protoplasts without the phosphatase overexpression. Moreover, we showed that ABI1 plays an opposite role to SnRK2.4 in regulation of primary root (PR) growth under salinity stress; SnRK2.4 is involved in positive regulation of PR growth of plants exposed to salinity [22], whereas ABI1 in negative regulation, according to our results presented here.

There are several data indicating an ABA-independent mechanism of PR growth regulation under salt stress conditions. Duan et al. [46] showed that NaCl shock significantly activated ABA signaling only in lateral roots (LRs) and not in PRs. Moreover, they showed that PR growth was similarly affected by salt in mutants of ABA synthesis and ABA signaling as in WT plants, in contrast to the growth inhibition of LRs. These facts, as well as data presented by McLoughlin et al. [22] and our results point to the involvement of SnRK2.4 and ABI1 in an ABA-independent pathway regulating PR growth under salinity. However, we cannot rule out the possibility that ABI1 regulates root growth under salt stress also through dephosphorylation of other proteins. It should be kept in mind that clade A PP2Cs have many more targets. They interact also with calcium dependent protein kinases, CPKs [47, 48], and with several other proteins, among them downstream elements of SnRKs and CPKs (e.g., transcription factors [47]). The fact that clade A PP2Cs regulate various signaling pathways in plants at multiple levels indicates that they constitute a hub in the cross talk between ABA-dependent and ABA-independent stress and development signaling pathways in plants.

Numerous targets of clade A PP2Cs and their functional redundancy make the studies on their role in the regulation of particular protein kinases in physiological context challenging. Moreover, the question still remains of how the activity of the phosphatases is regulated in response to various environmental stresses not involving a direct increase in ABA level. So far, PYR/PYL/RCAR ABA receptors are known to regulate the activity of clade A PP2Cs in response to ABA. It was proposed that the phosphatase activity is also inhibited by their interaction with certain PYR/PYL/RCAR receptors in an ABA-independent manner [49]. Particularly, PYL10 was reported to inhibit PP2C activity in an ABA-independent manner. However, this result has been recently refuted by Li et al. [50], who demonstrated that PYL10 activity is ABA-dependent. Moreover, when phosphorylated protein substrates (not small phosphopeptides) have been tested as substrates of PP2Cs, followed by direct analysis of their phosphorylation status, no substantial ABA-independent inhibition by ABA receptors could be found [51, 52]. Data published by Vlad et al. [25] indicate that in response to osmotic stress SnRK2.6 can be regulated by clade A PP2Cs, since kinase activity was notably increased in a *hab1-1 abi1-2 abi2-2* triple loss-of-function mutant compared to wild type. However, activation of SnRK2.6 by osmotic stress occurred in ABA-insensitive mutants carrying specific PP2C mutations that make them refractory to inhibition by PYR/PYL/RCAR proteins [25]. This suggests that ABA receptors are rather not involved in the negative regulation of the PP2Cs in response to osmotic stress. Possibly, other cellular partners of PP2Cs exist which inhibit their activity in an ABA-independent manner. One example of such proteins are calcineurin B-like proteins (CBLs). Lan et al. [53] showed that some CBLs interact with and inhibit PP2CA and in this way restore the activity of CBL-interacting protein kinase 6 (CIPK6) and the CIPK6-dependent activation of the K^+^ inward rectifying channel AKT1 in plant cells. Nevertheless, extensive studies are needed to elucidate the mechanism triggering the ABA-independent inhibition of PP2C phosphatases in response to osmotic stress. Not only proteins can be considered as potential inhibitors of phosphatases in response to osmotic stress. Very tempting is the idea that the phosphatases involved in the regulation of the SnRK2 family members are inactivated by various second messengers produced transiently in early steps of stress response, e.g., reactive oxygen species (ROS), phosphatidic acid (PA), or nitric oxide (NO). It has been shown that PA and ROS inhibit some of PP2Cs [54–56]. However, there are data showing that reagents inducing oxidative stress [57] and S-nitrosylation [58] also inhibit SnRK2s. Since NO and ROS can regulate phosphatases as well as kinases, signal transduction most probably relies on timing and strength of the signal.

Moreover, we cannot exclude the possibility that, in contrast to a fast response, SnRK2s from group 1 might be involved in ABA-dependent signaling in sustained stress conditions and that other PP2Cs are also involved in regulation of kinases from group 1 of the SnRK2 family.

Additionally, our results show that apart from PP2Cs, the activity of SnRK2.4/SnRK2.10 seems likely to be regulated by OA-sensitive Ser/Thr phosphoprotein phosphatases from the PPP family. SnRK2s belong to the AMPK/SNF1 family. The activity of mammalian AMP-activated protein kinase (AMPK) and yeast sucrose non-fermenting 1 (SNF1) kinase is negatively regulated by several protein phosphatases: type 2A (PP2A), PP1, and PP2C [59–63]. Numerous reports link plant PPPs with osmotic stress signaling and defense responses [64–71]. The published data suggest that the phosphatases can play both positive and negative roles in osmotic stress and ABA signal transduction. Recently it was shown that several PP2A regulatory subunits interact with SnRK2.6/OST1, as well as with other ABA-activated SnRK2s [72]. Surprisingly, the authors did not observe any significant cross-regulation of PP2A and SnRK2 activity in plants exposed to ABA. Moreover, while this paper was under revision, Hou et al. [73] showed that Type One Protein Phosphatase 1 (TOPP1) and its regulatory protein, At Inhibitor-2 (AtI-2), negatively regulate ABA-activated SnRK2s and subsequently ABA signaling. It is challenging to determine which PPP phosphatase(s) is involved in the regulation of ABA-non-activated SnRK2s in response to osmotic stress, first of all because of the scarce information on the physiological roles of these phosphatases, but also because of their complexity. The plant PPP family comprises of PP1, PP2A, PP4, PP5, PP6, PP7, Kelch-like repeat domain (PPKLs) and Shewanella-like protein (SLP) phosphatases (for review see Uhrig et al., [74]); OA affects all of them except SLPs. The PP2A holoenzyme consists of three types of subunits—catalytic (C), scaffolding/regulatory (A), and regulatory (B), of which five, three and seventeen isoforms, respectively, have been identified in Arabidopsis. They form different holoenzymes with divergent functions. Several regulatory subunits have also been identified for PP4 and PP6. Interestingly, the PP6 phosphatase catalytic subunits can interact with RCN1 (roots curl in n-naphthylphthalamic acid 1), a regulatory subunit of PP2A [75], which makes the PPP regulation even more complicated. Similar complexity also concerns PP1 phosphatases; there are nine PP1s in Arabidopsis, and numerous regulatory subunits. Identification of phosphatases belonging to the PPP family, which are involved in the inhibition of ABA-non-activated SnRK2s, is currently under investigation in our laboratory.

## Conclusions

Our results revealed that ABI1 plays a wide role in SnRK2s regulation, inhibiting not only the ABA-activated members of the family, but also SnRK2.4, a kinase not activated in response to ABA. Moreover, it seems that ABI1 is not the only phosphatase, which regulates the activity of SnRK2s activated by osmotic stress in an ABA-independent manner; members of the PPP family are also involved in such regulation. It remains to be investigated how ABI1 and also other SnRK2 phosphatases are inhibited in response to osmotic stress independently of ABA to allow transient SnRK2 activation.

## Methods

### Plant material and growth conditions

*Arabidopsis thaliana* lines used: ecotype Col-0 *-* wild type; T-DNA insertion lines: *abi1td* (corresponding to *abi1-3*) (SALK_076309, [76]) and *snrk2.4* (SALK_080588, [22]). Seeds were sterilized as described in Kulik et al. [77]. Plants were grown as described in McLoughlin et al. [22]. Arabidopsis T87 cells were maintained as described by Yamada et al. [78] and treated with 500 mM NaCl six days after subculturing. For okadaic acid (OA) treatment cells were preincubated with 0.2 μM or 1 μM OA (Enzo Life Sciences, www.enzolifesciences.com) or 0.8 % ethanol as control for 2 h and then treated with 500 mM NaCl for indicated time.

Root growth assay was performed as described in McLoughlin et al. [22]. Change in root length was determined using the ImageJ software (http://imagej.nih.gov/ij/). *T*-test performed in Sigma Plot for Windows Version 13.0 software was applied for statistical analysis.

### Yeast two-hybrid interaction assays

Construction of pGBT9 plasmids with cDNA of SnRK2.4, SnRK2.6 and SnRK2.8 was described previously [79]. The cDNA encoding SnRK2.10 or PP2C phosphatases were cloned into pGBT9 or pGAD424 (Clontech, www.clontech.com), respectively, primers used are listed in Additional file 6. The AH109 yeast strain was transformed according to [80]. Transformants were selected on SD medium without Leu/Trp and then assayed on SD medium without Leu/Trp/His supplemented with 1 mM - 10 mM of aminotriazole (3-AT) or SD without Leu/Trp/Ade.

### Expression and purification of recombinant proteins

Recombinant SnRK2.4, SnRK2.6 and SnRK2.8 were prepared as described previously [79]. Plasmid encoding ABI1 was obtained by PCR amplification of cDNA of ABI1 and its insertion into the pGEX-4 T-1 vector (GE Healthcare, www.gelifesciences.com). The pGEX-AP2C3 plasmid [81] was kindly provided by Dr. Irute Meskiene (Max F. Perutz Laboratories, University of Vienna). Recombinant PP2Cs were produced in *E. coli* Rosetta (Novagen) at 37 °C for 2 h and purified according to Frangioni et al. [82].

### Preparation of plant protein extracts

Protein extracts were prepared as described previously [77].

### Immunoblot analysis

Antibodies used: anti-SnRK2.4/SnRK2.10 (previously named anti-NtOSAK/SnRK2.4/SnRK2.10), described in Kulik et al. [77], and anti-P-SnRK2 phospho-specific polyclonal raised against the phosphopeptide KS(P)TVGT) (BioGenes GmbH, www.biogenes.de), described in Burza et al. [21]; anti-GFP (Molecular Probes, www.thermofisher.com); anti-c-Myc (Santa Cruz Biotechnology, www.scbt.com); StrepMAB-Classic, HRP conjugate (IBA GmbH, www.iba-lifesciences.com). Western blotting with anti-P-SnRK2 and anti-SnRK2.4/SnRK2.10 antibodies was performed as described previously [21, 77], whereas for anti-GFP, anti-c-Myc and anti-StrepTag as indicated by the manufacturers.

### Immunoprecipitation

Immunoprecipitation was performed as described previously [77], with minor modifications. For every mg of protein from crude extract 50 μL of protein A-agarose slurry (Santa Cruz Biotechnology, www.scbt.com) and 100 μg of antibodies was used. For analysis of SnRK2.4/SnRK2.10 activity immunoprecipitation was performed from 500 μg of crude protein extract.

### In-gel kinase assay

In-gel kinase activity assays were performed according to Zhang and Klessig [83].

### In vitro phosphatase assays

In vitro dephosphorylation assays with SnRK2s as substrates were performed in buffer containing 20 mM Tris–HCl, pH 7.5 and 25 mM MgCl_2_ (final volume 25 μL)_,_ for 30 min at 30 °C. Reactions were stopped by addition of Laemmli sample buffer and boiling for 2 min. Specific activity of recombinant phosphatases was determined using the Serine/Threonine Phosphatase Assay System (Promega, www.promega.com) and activity units (U) were calculated for each phosphatase, where one U is the amount of phosphatase, which releases 1 picomole of phosphate per minute.

### In vitro binding assay

Glutathione-Sepharose 4B beads (20 μL) (GE Healthcare, www.gelifesciences.com) with bound GST-ABI1 or GST were incubated with 10 μg of recombinant SnRK2.4 in binding buffer (50 mM Tris–HCl, pH 8.0, 50 mM NaCl, 0.01 % Triton X-100, 40 mM DTT and 1x Complete Protease Inhibitor Cocktail Roche, Sigma-Aldrich, www.sigmaaldrich.com) in a final volume of 100 μL. After 45 min of shaking at RT the beads were washed 5 times with the binding buffer. Proteins attached to the resin were separated by SDS-PAGE, and either visualized by Coomassie staining, or transferred onto PVDF membrane and visualized by immunoblotting with anti-SnRK2.4/SnRK2.10 antibodies.

### Protoplast transient expression assay

For expression of proteins in protoplasts followed by analysis of enzyme activity, pSAT vectors were used [84]. Construction of pSAT6-EGFP-C1 plasmids with cDNA of SnRK2.4 and SnRK2.8 was described previously [79]. For generation of c-Myc-ABI1 the cDNA encoding the phosphatase was cloned into a modified pSAT6-MCS vector, with the c-Myc coding sequence introduced between the NcoI and BglII sites. Protoplasts from T87 cells were isolated and transformed as described previously [79]. Constructs for BiFC assays were prepared using the Gateway®Cloning system (Invitrogen, www.thermofisher.com). ABI1, SnRK2.4, SnRK2.6 and SnRK2.8 cDNAs were PCR amplified and cloned into the pENTR®-D/TOPO™ vector. Next, the cDNAs were introduced into pSITE II n-EYFP-N1 or pSITE II c-EYFP-C1 vectors [85] by Gateway LR reaction. Protoplasts from leaves of 5-week-old Arabidopsis Col-0 plants were isolated and transformed according to [86]. Approximately 1 x 10^6^ protoplasts were transfected with 40 μg of plasmid DNA, 20 μg of each plasmid. Sixteen hours after transformation YFP signal was visualized by confocal microscopy as described previously [79] or the protoplasts were treated with 300 mM NaCl for time indicated in Results.

### Remaining accession numbers

Sequence data for the following can be found in the TAIR database: HAB1, At1g72770; HAB2, At1g17550; AHG1, At5g51760; HAI1, At5g59220; HAI2, At1g07430; HAI3, At2g29380.

## Abbreviations

ABA, abscisic acid; ABI1, ABA-insensitive 1; AHG3, ABA-hypersensitive germination 3; AMPK, AMP-activated protein kinase; ARK, ABA and abiotic stress-responsive Raf-like kinase; AtI-2, At inhibitor-2; BiFC, bimolecular fluorescence complementation; CBL, calcineurin B-like proteins; CIPK, CBL-interacting protein kinase; CPK, calcium-dependent protein kinase; GSK3, glycogen synthase kinase 3; HAB1, hypersensitive to ABA 1; LR, lateral root; NO, nitric oxide; NtOSAK, *Nicotiana tabacum* osmotic stress-activated kinase; OA, okadaic acid; PA, phosphatidic acid; PP2C, phosphoprotein phosphatase 2C; PPKL, protein phosphatase with Kelch-like domain; PPP, phosphoprotein phosphatases; PR, primary root; PYL, PYR1-like; PYR1, pyrabactin resistance 1; RCAR, regulatory component of ABA receptor; RCN1, roots curl in n-naphthylphthalamic acid 1; ROS, reactive oxygen species; SLP, Shewanella-like protein phosphatase; SnRK2, SNF1-related protein kinase 2; TOPP1, type one protein phosphatase 1; YTH, yeast two-hybrid.

## Additional files

Additional file 1: Figure S1. Phylogenetic tree of Arabidopsis (SnRK2.1 to SnRK2.10), rice (SAPK1 to SAPK10) SnRK2s, and tobacco SnRK2 (NtOSAK). (PDF 427 kb) Additional file 2: Figure S2. Inhibition of NtOSAK activity by GST-NtPP2C2 is correlated with dephosphorylation of Ser-154 and Ser-158 in the kinase activation loop. (PDF 683 kb) Additional file 3: Figure S3. Activity of recombinant PP2Cs used in this study. (PDF 165 kb) Additional file 4: Figure S4. GST does not affect SnRK2 kinase activity in in vitro assay. (PDF 251 kb) Additional file 5: figure S5. Primary root growth inhibition of studied plant lines under salt stress. (PDF 229 kb) Additional file 6: Table S1. PCR primers used for cloning. (DOC 53 kb) Additional file 7: Supporting Methods. (DOC 34 kb)
