# Supplementary material for: Phosphatase ABI1 and okadaic acid-sensitive phosphoprotein phosphatases inhibit salt stress-activated SnRK2.4 kinase
Source: BMC Plant Biol. 2016 Jun 13;16:136. doi: 10.1186/s12870-016-0817-1 (PMC4907068; doi:10.1186/s12870-016-0817-1)
Supplement: Additional file 1: Figure S1. — Phylogenetic tree of Arabidopsis (SnRK2.1 to SnRK2.10), rice (SAPK1 to SAPK10) SnRK2s, and tobacco SnRK2 (NtOSAK). (PDF 427 kb) [file 12870_2016_817_MOESM1_ESM.pdf]

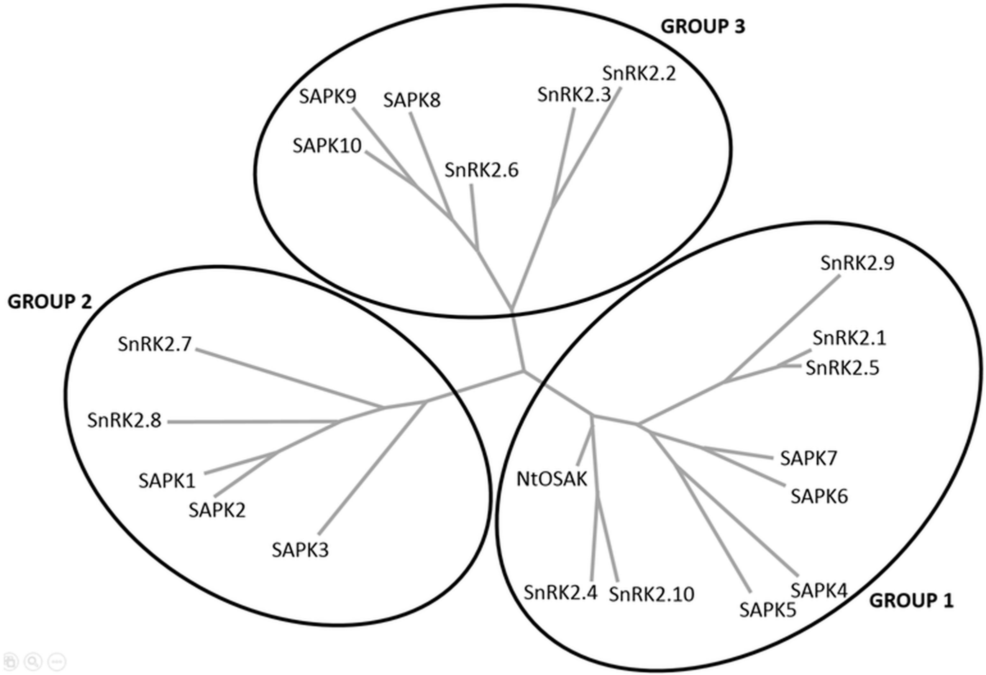

Figure S1. Phylogenetic tree of Arabidopsis (SnRK2.1 to SnRK2.10), rice (SAPK1 to SAPK10) SnRK2s, and tobacco SnRK2 (NtOSAK).
Group 1 - ABA-non-activated kinases, group 2 - kinases non-activated or weakly activated by ABA, group 3 - ABA-activated kinases. The tree was built using Phylogeny.fr platform (http://phylogeny.lirmm.fr/)
[Dereeper A.*, Guignon V.*, Blanc G., Audic S., Buffet S., Chevenet F., Dufayard J.F., Guindon S., Lefort V., Lescot M., Claverie J.M., Gascuel O. *Phylogeny.fr: robust phylogenetic analysis for the non-specialist*. Nucleic Acids Res. 2008 Jul 1;36(Web Server issue):W465-9. Epub 2008 Apr 19]
