# Supplementary material for: Phosphatase ABI1 and okadaic acid-sensitive phosphoprotein phosphatases inhibit salt stress-activated SnRK2.4 kinase
Source: BMC Plant Biol. 2016 Jun 13;16:136. doi: 10.1186/s12870-016-0817-1 (PMC4907068; doi:10.1186/s12870-016-0817-1)
Supplement: Additional file 2: Figure S2. — Inhibition of NtOSAK activity by GST-NtPP2C2 is correlated with dephosphorylation of Ser-154 and Ser-158 in the kinase activation loop. (PDF 683 kb) [file 12870_2016_817_MOESM2_ESM.pdf]

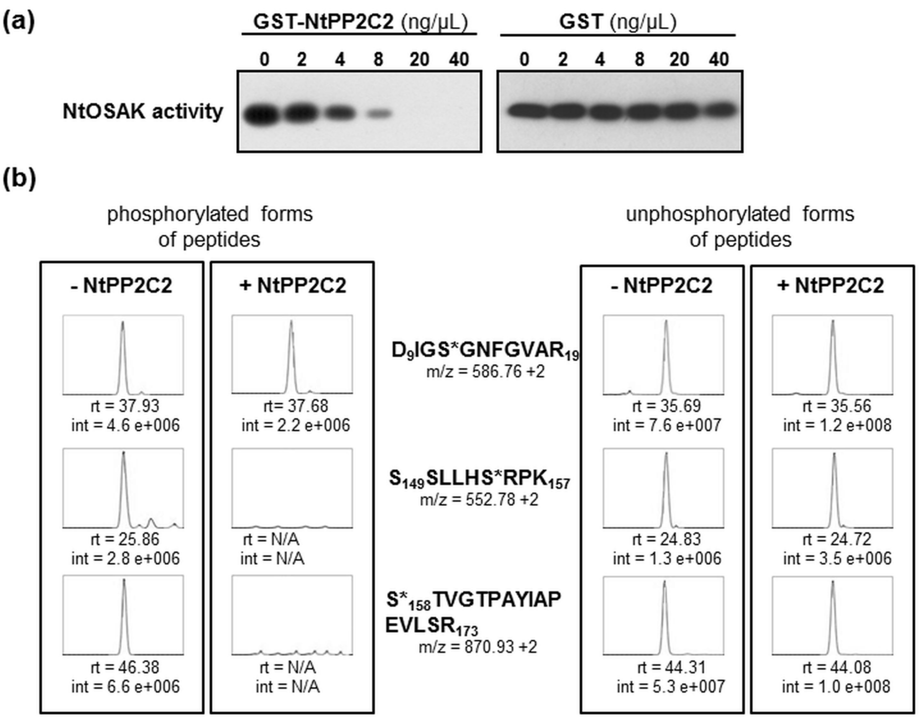

Figure S2. Inhibition of NtOSAK activity by GST-NtPP2C2 is correlated with dephosphorylation of Ser-154 and Ser-158 in the kinase activation loop.

(a) Equal amounts of active NtOSAK immunoprecipitated from BY-2 cells were pre-incubated with indicated amounts of GST-NtPP2C2, or GST as control and the kinase activity was analyzed by in-gel kinase assay using MBP as substrate.

(b) Immunoprecipitated NtOSAK was pre-incubated with NtPP2C2 (+ NtPP2C2) or not (- NtPP2C2) and phosphorylated NtOSAK-specific peptides were identified by LC/MS. Graphs represent chromatographic peaks corresponding to peptides either in phosphorylated (left panels) or unmodified (right panels) form. Phosphorylated residues are marked with an asterisk. Data for two peptides (S_{149}SLLHS*RPK_{157} and S_{158}*TVGTPAYIAPEVLSR_{173}) localized in the kinase activation loop and one (D_9IGS*GNFGVAR_{19}) outside this region are presented. For each of the signals its LC retention time and intensity (arb. units) are shown. The identity of the peptide was reconfirmed by analysis of fragmentation spectra in each of the experiments shown.
