# Supplementary material for: Phosphatase ABI1 and okadaic acid-sensitive phosphoprotein phosphatases inhibit salt stress-activated SnRK2.4 kinase
Source: BMC Plant Biol. 2016 Jun 13;16:136. doi: 10.1186/s12870-016-0817-1 (PMC4907068; doi:10.1186/s12870-016-0817-1)
Supplement: Additional file 3: Figure S3. — Activity of recombinant PP2Cs used in this study. (PDF 165 kb) [file 12870_2016_817_MOESM3_ESM.pdf]

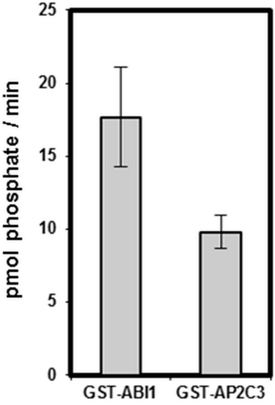

Figure S3. Activity of recombinant PP2Cs used in this study. Activity of 2 µg of each recombinant phosphatase was measured using the Serine/Threonine Phosphatase Assay System (Promega). The results were used for calculation of the amount of enzyme releasing 1 picomole of phosphate per minute (U) for each of the phosphatases. Error bars indicate SD (n = 3).
