# Supplementary material for: Phosphatase ABI1 and okadaic acid-sensitive phosphoprotein phosphatases inhibit salt stress-activated SnRK2.4 kinase
Source: BMC Plant Biol. 2016 Jun 13;16:136. doi: 10.1186/s12870-016-0817-1 (PMC4907068; doi:10.1186/s12870-016-0817-1)
Supplement: Additional file 4: Figure S4. — GST does not affect SnRK2 kinase activity in in vitro assay. (PDF 251 kb) [file 12870_2016_817_MOESM4_ESM.pdf]

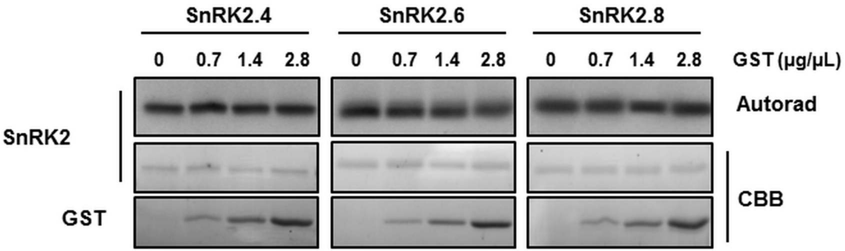

Figure S4. GST Does Not Affect SnRK2 Kinase Activity in *In Vitro* Assay.
Recombinant SnRK2s (1 µg each) were incubated with increasing amounts of GST (corresponding to the amounts of GST-fused phosphatases used in assays presented in Fig 3) in a final volume of 25 µl, for 30 min at 30°C and kinase activity was analyzed by in-gel kinase assay using MBP as substrate. Autorad, autoradiograph; CBB, Coomassie Brilliant Blue.
