# Supplementary material for: Phosphatase ABI1 and okadaic acid-sensitive phosphoprotein phosphatases inhibit salt stress-activated SnRK2.4 kinase
Source: BMC Plant Biol. 2016 Jun 13;16:136. doi: 10.1186/s12870-016-0817-1 (PMC4907068; doi:10.1186/s12870-016-0817-1)
Supplement: Additional file 5: figure S5. — Primary root growth inhibition of studied plant lines under salt stress. (PDF 229 kb) [file 12870_2016_817_MOESM5_ESM.pdf]

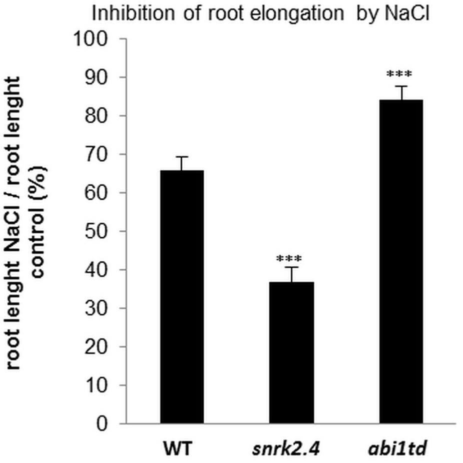

Figure S5. Primary root growth inhibition of studied plant lines under salt stress.
Seven-day-old seedlings grown vertically on ½ MS media were transferred into square Petri plates with control medium or medium with 115 mM NaCl. After seven additional days root growth inhibition was calculated as % of root lenght of plants grown on medium with salt to those grown on control medium. The graphs presents mean values (± SE), n=7. Statistical analysis was done by Mann-Whitney Rank Sum Test.
