# Supplementary material for: Phosphatase ABI1 and okadaic acid-sensitive phosphoprotein phosphatases inhibit salt stress-activated SnRK2.4 kinase
Source: BMC Plant Biol. 2016 Jun 13;16:136. doi: 10.1186/s12870-016-0817-1 (PMC4907068; doi:10.1186/s12870-016-0817-1)
Supplement: Additional file 6: Table S1. — PCR primers used for cloning. (DOC 53 kb) [file 12870_2016_817_MOESM6_ESM.doc]

**Table S1.** PCR primers used for cloning

| **Gene** | **Primer name** | **Sequence (5’-3’)** | **vector** |
| --- | --- | --- | --- |
| *c-Myc* | mycsat3F | TTTTCCATGGAACAGAAACTGATCTCTGAAGAAGATCTGATCTCGAGTTT | pSAT6-MCS |
|  | mycsat3R | AAACTCGAGATCAGATCTTCTTCAGAGATCAGTTTCTGTTCATGGAAAA |  |
| *NtPP2C2* | Np2c2pGF | TAAGGATCCATGGCTGGGATGTGTTGTGG | pGEX4T3 |
|  | Np2c2s1R | ATTCCCGGGTTACAAATCTTTTCTTAAATCAACCAC |  |
| *ABI1* | ABIsat3F | TTTAAGCTTCATGGAGGAAGTATCTCCGGC | c-Myc-pSAT6 |
|  | ABIsat3R | AATCCCGGGTCAGTTCAAGGGTTTGCTC |  |
| *SnRK2.4* | 24pEntF | CACCATGGACAAGTACGAGCTGGTG | pENTR®-D/TOPO™ |
|  | 24pEntR | TCAACTTATTCTCACTTCTCCACTTG |  |
| *SnRK2.6* | 26pEntF | CACCATGGATCGACCAGCAGTGAGTG |  |
|  | 26pEntR | TCACATTGCGTACACAATCTCTCCG |  |
| *SnRK2.8* | 28pEntF | CACCATGGAGAGGTACGAAATAGTG |  |
|  | 28pEntR | TCACAAAGGGGAAAGGAGATCAG |  |
| *ABI1* | ABIpEntF | CACCATGGAGGAAGTATCTCCGGCG |  |
|  | ABIpEntR | GTTCAAGGGTTTGCTCTTGAGTTTC |  |
| SnRK2.10 | F-SnRK2.10 | TCCGAATTCATGGACAAGTACGAGCTTGTT | pGBT9 |
|  | R-SnRK2.10 | TTTGTCGACTTAACTGACTCGGACTTCTCC |  |
| HAI1 | F2HSHAI1 | ATAGAATTCATGGCTGAGATTTGTTACGAGAACGAG | pGAD424 |
|  | R2HSHAI1 | TTTCTCGAGCTACGTGTCTCGTCGTAGAAC |  |
| HAI2 | FPP2CA4 | ACGGAATTCATGGCGGATATTTGTTATGAA | pGAD424 |
|  | RPP2CA4 | AGCCTGCAGTCAAGCAACGTGTCTCTTTCT |  |
| HAI3 | FPP2CA5 | TACCCGGGTATGGCCGAGATATGTTACGAA |  |
|  | R-PP2CA5 | TCGCTGCAGTTATCTTCTGAGATCAATCAC |  |
| HAB1 | FHAB1 | ACAGAATTCATGGAGGAGATGACTCCCGCA |  |
|  | RHAB1 | AGCGTCGACTCAGGTTCTGGTCTTGAACTT |  |
| HAB2 | F-HAB2 | TTTGAATTCATGGAAGAGATTTCACCTGCAG |  |
|  | R-HAB2 | ATCGTCGACTCAAGATCTGGTCTTGAACTTTC |  |
| AHG1 | F2HSAHG1 | ATACAATTGATGACTGAAATCTACAGAACAATTTC |  |
|  | R2HSAHG1 | TTTGTCGACTTACTGAGAGCTATTCTTGAG |  |
| AHG3 | AHG3-AD | TACCCGGGTATGGCCGAGATATGTTACGAA |  |
|  | RAHG3 | CCCGTCGACTTAAGACGACGCTTGATTATT |  |
| ABI1 | FABI1 | TAAGGATCCAAATGGAGGAAGTATCTCCGGCG |  |
|  | RABI1 | ACCGTCGACTCAGTTCAAGGGTTTGCTCTT |  |
| ABI2 | FABI2 | AACCCGGGAATGGACGAAGTTTCTCCTGCA |  |
|  | RABI2 | CCCCTCGAGTCAATTCAAGGATTTGCTCTT |  |
