# Supplementary material for: Phosphatase ABI1 and okadaic acid-sensitive phosphoprotein phosphatases inhibit salt stress-activated SnRK2.4 kinase
Source: BMC Plant Biol. 2016 Jun 13;16:136. doi: 10.1186/s12870-016-0817-1 (PMC4907068; doi:10.1186/s12870-016-0817-1)
Supplement: Additional file 7: — Supporting Methods. (DOC 34 kb) [file 12870_2016_817_MOESM7_ESM.doc]

**SUPPORTING METHODS**

**Expression and purification of recombinant GST-NtPP2C2**

Plasmid encoding NtPP2C2 was obtained by PCR amplification of cDNA of NtPP2C2 and its insertion into pGEX-4T-3 vector (GE Healthcare, [www.gelifesciences.com](http://www.gelifesciences.com/)). Recombinant protein was produced in *E. coli* Rosetta Novagen at 37°C for 2 h and purified according to .

**Analysis of NtOSAK activity and its phosphorylation status after GST-NtPP2C2** **treatment**

For small-scale dephosphorylation reactions with native NtOSAK as substrate the kinase was immunoprecipitated from 300 µg of crude protein extract. For large-scale dephosphorylation reactions followed by MS analysis NtOSAK was immunoprecipitated from 1.5 mg of crude protein extract. For every mg of protein from crude extract 50 µL of protein A-agarose slurry (Santa Cruz Biotechnology) was incubated for 2 h with 100 µg of anti-NtOSAK antibodies. To assess the impact of GST-NtPP2C2 on the activity of immunoprecipitated NtOSAK, increasing amounts of the phosphatase (0, 2, 4, 8, 20, and 40 ng/µL), or GST as control, were added to the samples to a final volume of 25 µL. After incubation, NtOSAK activity was analyzed by in-gel kinase assay. For assays followed by MS analysis, 50 ng/µL of GST-NtPP2C2 was added to the sample with immunoprecipitated NtOSAK, to a final volume of 50 µL. The phosphorylation status of phosphopeptides from the kinase activation loop, namely S_149_SLLHS*RPK_157_ and S*_158_TVGTPAYIAPEVLSR_173_, and phosphopeptide D_9_IGS*GNFGVAR_19_, localized outside the kinase activation loop, was tested by mass spectrometry. Phosphorylation sites were determined basically as described previously (Burza et al. 2006), with minor modifications; after trypsin digestion peptides were applied to RP-18 precolumn (nanoACQUITY Symmetry C18; Waters, www.waters.com) and then transferred to nano-HPLC RP-18 column (nanoACQUITY BEH C18; Waters, www.waters.com) using an acetonitrile gradient (0–60% AcN in 40 min) in the presence of 0.05% formic acid with the ﬂow rate of 150 nL/min.
